# Molecular prevalence, genetic characterization and patterns of *Toxoplasma gondii* infection in domestic small mammals from Cotonou, Benin[Fn FN1]

**DOI:** 10.1051/parasite/2022058

**Published:** 2022-12-21

**Authors:** Jonas R. Etougbétché, Azra Hamidović, Henri-Joël Dossou, Maeva Coan-Grosso, Roxane Roques, Nicolas Plault, Gualbert Houéménou, Sylvestre Badou, Antoine A. Missihoun, Issaka Youssao Abdou Karim, Lokman Galal, Christophe Diagne, Marie-Laure Dardé, Gauthier Dobigny, Aurélien Mercier

**Affiliations:** 1 Ecole Polytechnique d’Abomey-Calavi, Laboratoire de Recherche en Biologie Appliquée, Unité de Recherche sur les Invasions Biologiques, Université d’Abomey-Calavi 01 BP 2009 Cotonou Benin; 2 Laboratoire de Génétique Moléculaire et d’Analyse des Génomes, Faculté des Sciences et Techniques, Université d’Abomey-Calavi 01BP 526 Cotonou Bénin; 3 Inserm U1094, IRD U270, Univ. Limoges, CHU Limoges, EpiMaCT - Epidémiologie des maladies chroniques en zone tropicale, Institut d’Epidémiologie et de Neurologie Tropicale, Omega Health 87000 Limoges France; 4 Institut du Cadre de Vie (ICaV), Université d’Abomey-Calavi BP 2899 Abomey-Calavi Benin; 5 Ecole Polytechnique d’Abomey-Calavi, Laboratoire de Biotechnologie Animale et de Technologie des Viandes, Université d’Abomey-Calavi 01 BP 2009 Cotonou Benin; 6 Institut de Recherche pour le Développement, UMR CBGP (IRD, INRA, Cirad, Montpellier SupAgro), Montpellier Université d’Excellence 755 avenue du campus Agropolis 34988 Montferrier-sur-Lez Cedex France; 7 Centre National de Référence (CNR) Toxoplasmose/Toxoplasma Biological Center (BRC), Centre Hospitalier-Universitaire Dupuytren 87000 Limoges France; 8 Unité Peste, Institut Pasteur de Madagascar BP 1274 Ambatofotsikely Avaradoha 101 Antananarivo Madagascar

**Keywords:** *Toxoplasma gondii*, Small mammals, Parasite ecology, Infectious disease, Molecular epidemiology, Urban eco-epidemiology, Benin

## Abstract

Toxoplasmosis, one of the most prevalent parasitic infections in humans and animals, is caused by the intracellular protozoan parasite *Toxoplasma gondii*. Small mammals play a key role as intermediate reservoir hosts in the maintenance of the *T. gondii* life cycle. In this study, we estimated the molecular prevalence and provide genetic diversity data for *T. gondii* in 632 small mammals sampled in four areas of Cotonou city, Benin. Both the brain and heart of each individual were screened through *T. gondii*-targeting qPCR, and positive samples were then genotyped using a set of 15 *T. gondii*-specific microsatellites. Prevalence data were statistically analyzed in order to assess the relative impact of individual host characteristics, spatial distribution, composition of small mammal community, and urban landscape features. An overall *T. gondii* molecular prevalence of 15.2% was found and seven genotypes, all belonging to the *Africa 1* lineage, could be retrieved from the invasive black rat *Rattus rattus* and the native African giant shrew *Crocidura olivieri*. Statistical analyses did not suggest any significant influence of the environmental parameters used in this study. Rather, depending on the local context, *T. gondii* prevalence appeared to be associated either with black rat, shrew, or mouse abundance or with the trapping period. Overall, our results highlight the intricate relationships between biotic and abiotic factors involved in *T. gondii* epidemiology and suggest that *R. rattus* and *C. olivieri* are two competent reservoirs for the *Africa 1* lineage, a widespread lineage in tropical Africa and the predominant lineage in Benin.

## Abstract (fr)

La toxoplasmose, l’une des infections parasitaires les plus répandues chez l’homme et les animaux, est causée par le parasite protozoaire intracellulaire *Toxoplasma gondii*. Les petits mammifères jouent un rôle clé en tant qu’hôtes réservoirs intermédiaires dans le maintien du cycle de vie de *T. gondii*. Dans cette étude, nous estimons sa prévalence moléculaire et fournissons des données sur sa diversité génétique chez 632 petits mammifères échantillonnés dans quatre localités de la ville de Cotonou. Le cerveau et le cœur de chaque individu ont été analysés par qPCR ciblant *T. gondii*, et les échantillons positifs ont ensuite été génotypés à l’aide d’un ensemble de 15 microsatellites spécifiques à *T. gondii*. Les données de prévalence ont été analysées statistiquement afin d’évaluer l’impact relatif des caractéristiques individuelles de l’hôte, de la distribution spatiale, de la composition de la communauté des petits mammifères ainsi que des caractéristiques du paysage urbain. Une prévalence moléculaire globale de *T. gondii* de 15,2 % a été estimée et sept génotypes, tous appartenant à la lignée *Africa 1,* ont pu être extraits du rat noir *Rattus rattus*, espèce envahissante, et de la musaraigne *Crocidura olivieri*, espèce indigène. Les analyses statistiques n’ont pas suggéré d’influence significative des paramètres environnementaux utilisés dans cette étude. Au contraire, selon le contexte local, la prévalence de *T. gondii* semble être associée à l’abondance de rats noirs, de musaraignes ou de souris ainsi qu’à la période de piégeage. Dans l’ensemble, nos résultats mettent en évidence les relations complexes entre les facteurs biotiques et abiotiques impliqués dans l’épidémiologie de *T. gondii* et suggèrent que *R. rattus* et *C. olivieri* sont deux réservoirs compétents pour la lignée *Africa 1*, une lignée répandue en Afrique tropicale et prédominante au Bénin.

## Introduction

Small mammals, including rodents, have an important epidemiological role in the evolutionary ecology and transmission cycle of *Toxoplasma gondii*, for which they act as natural reservoirs. They are one of the preferred prey groups of the definitive felid hosts (e.g., cats [14, 35]), and therefore play a key role in the circulation of *Toxoplasma* strains within local and novel environments [30, 42, 62, 67]. This is particularly true in the increasingly changing global context that favors the ongoing introduction and spread of invasive rodents [40, 41]. Accordingly, rodents are generally considered relevant hosts to assess environmental contamination by toxoplasmosis, and to estimate the infection risk for definitive hosts [1, 6, 67]. Nevertheless, considering rodents a meaningful indicator to predict environmental contamination by *T. gondii* requires us to differentiate between potential biological and ecological differences within and between reservoir rodent species. As an illustration, a positive relationship between rodent body mass and probability to be infected by *T. gondii* has been found both within (i.e., older individuals were more often infected [20, 67]) as well as between species (i.e., larger species had a longer life span, hence more opportunities to encounter *T. gondii* [2, 20, 46]). Species-specific ecological requirements have also been found to influence *T. gondii* prevalence, essentially due to variation in oocyst–rodent contact patterns [68, 81]. For example, fossorial species that live in burrows are always in contact with soil and likely include *T. gondii* paratenic hosts (such as earthworms, [2]) in their diet. In addition to biological and ecological species-level features, the local environment is expected to influence the persistence of oocysts within the external environment, thus greatly impacting parasite eco-epidemiology [81, 84].

Despite this burgeoning knowledge on the reservoir-based ecology of *T. gondii*, the effects of environmental, ecological, and host-individual factors on the circulation of *T. gondii* in natural populations of rodent reservoirs have been scarcely investigated in a concomitant way. Moreover, studies on the prevalence of toxoplasmosis in small mammals sampled in various parts of the world show great variability following region and habitat, host species, and detection method [30]. In Africa, most studies are based on serological detection of *T. gondii* and seroprevalence was found to be relatively low: e.g., 1.6% and 1.2% in *Mus musculus* and *Mastomys natalensis*, respectively in Niamey, Niger [58]; 2.3% and 4.2% in *Rattus rattus* from rural Gabon [57] and across Senegal [11], respectively and 3.8% in *Rattus norvegicus* in Egypt [59]. However, such seroprevalence-based results have largely been questioned from several points of view, such as the intrinsic differences in host immune response [40] and the variable sensitivity/specificity of serological tests used [17, 30]. These limitations have therefore led to recognize qPCR-based approaches as more sensitive and reliable, since this method allows direct targeting of the parasite DNA in examined tissues [40].

Furthermore, while rodents, and specifically laboratory mice, are widely used to study toxoplasmosis in laboratory conditions (e.g., host immune response, parasite virulence and/or parasite amplification for subsequent genetic analyses), studies using the direct molecular approach for investigations in natural populations are largely lacking, especially in the African context as far as we are aware (but see e.g., [30, 40, 41]). However, investigations in natural populations are key to unravel the intricate eco-evolutionary processes underlying the circulation of *T. gondii* in various habitats. As an illustration, the first Types I, II and III isolates of this parasite were historically classified according to their virulence in the laboratory mouse [73]. However, such a virulence in laboratory mice is clearly not generalizable to all rodent species, and distinct resistance profiles have been observed in different subspecies or strains of the same rodent host species [49, 61]. These resistance profiles result from a complex evolutionary balance between the host immune response and the genetic diversity of *T. gondii* in each region of the world [22, 48, 69, 72].

The circulation of *T. gondii* in domestic animals has been demonstrated in several parts of Benin (West Africa), including Cotonou city [47, 82, 83], but no data are available for small mammals hosts. Nevertheless, this city has a major international seaport that has been shown to be responsible for the introduction of invasive rodent species and associated zoonotic pathogens into the country [13], thus questioning the genetic diversity and ecology of *T. gondii* strains that may circulate locally through these reservoir species. In this study, we explore the role of natural populations of small mammals (rodents and shrews) from Cotonou city in the epidemiology of *T. gondii.* To do so, we focus on the Cotonou small mammal community in order to *(i)* assess the molecular prevalence of *T. gondii* within each species-specific population, *(ii)* explore the impact of certain intrinsic and extrinsic factors on individual infection by *T. gondii*, and *(iii)* further document the genotypes circulating among small mammals within this large West African seaport city.

## Material and methods

### Ethics statement

Sampling was conducted under the research agreement signed between the Republic of Benin and the French Research Institute for Sustainable Development (IRD) renewed on April 6, 2017, as well as the scientific collaboration agreement between IRD and Abomey-Calavi University signed on the September 30, 2010 and renewed on July 3, 2019. All trapping sessions were conducted following the explicit oral authorization of local authorities (Cotonou City Hall and local heads of urban districts) as well as the agreement of each household’s owners and inhabitants. Field campaigns within the Cotonou Autonomous Seaport were implemented following official written authorization issued by its General Management Board. None of the species captured in this study have protection status (see CITES list, https://checklist.cites.org/). All animals were handled and processed in a humane manner, with respect to animal welfare following the guidelines of the American Society of Mammologists [74]. Our study and the uses of associated samples and genetic data were implemented following Nagoya protocol recommendations (Access and Benefit Sharing agreement number 608/DGEFC/DCPRN/PF-APA/SA).

### Study sites

**Figure 1:**
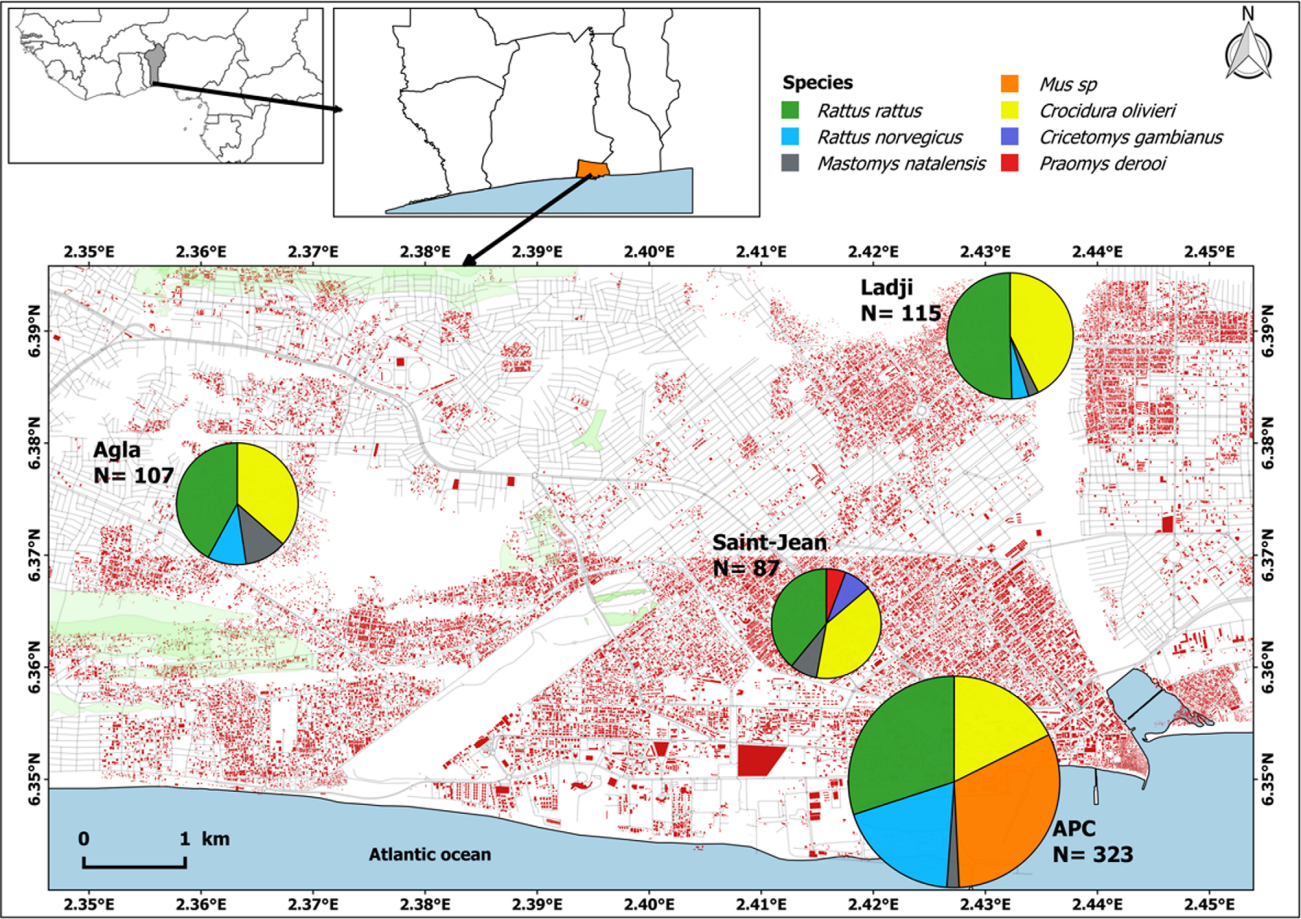
Distribution of sampling localities within Cotonou city, as well as micro-mammal trapping results. Circle sizes are proportional to the number of host individuals investigated for *T. gondii*.

Our study was performed across two major types of areas: *(i)* three socio-environmentally contrasted urban districts of Cotonou city between 2017 and 2018, namely Ladji, Agla and Saint-Jean; and *(ii)* Cotonou seaport (Autonomous Port of Cotonou, or APC) (Fig. 1). Though of rather recent origin, Agla is currently one of Cotonou’s most populous neighbourhoods. At the time of the present study, it was a still a partially swampy area undergoing rapid urbanization. It is located within a large shallow land and was thus characterized by wide flooded areas at the early stages of the rainy season, following accumulation of rainfall and elevation of the water table [52]. Ladji is located on the shore as well as upon Lake Nokoué (i.e., stilt-built houses). With over 10,000 inhabitants, this informal settlement is socio-environmentally very disadvantaged and suffers from insufficient basic services. It is partly flooded at the end of the rainy season following overflow of Lake Nokoué. Saint-Jean is a formal residential area of colonial origin. Even though the population is working-class, many services are formally available, and the area does not flood *per se* during the rainy season. However, wide ponds may persist in some places for days to a few weeks after heavy rains. APC is located in the central administrative highly human-made part of the city. Two traditional and seven industrial port zones were targeted: the former gather areas where traditional fishing products are disembarked, prepared, stored and sold, while the latter correspond to the canteens (where workers take lunch) as well as areas under restricted access (e.g., administrative services, docks, storehouses, workshops, garages, and parking lots) (for details about these APC sampling sites).

### Sampling

In each of the three districts, 9–11 households (hereafter designated “district sites”) were investigated (see details in [24, 26]), while nine sites were sampled in APC (hereafter designated “APC sites”). Each area was investigated twice: in October 2017 and June 2018 for Agla, Ladji, and Saint-Jean on the one hand, and in September-November 2017 and March 2018 for APC on the other. Standardized longitudinal trapping sessions were conducted for three consecutive nights using both Sherman and locally made wire mesh traps. A fish/peanut butter mixture was used as bait. Captured small mammals were brought alive into the laboratory where they were euthanized within the same day following diethyl-ether exposure and subsequent cervical dislocation, then sexed, weighed and measured. Immediately after euthanasia, presence of ectoparasitic fleas was checked by combing the animal. Particular attention was paid to unambiguous species-specific identification of all individuals through morphology and external measurements, cytochrome b DNA sequencing, PCR-RFLP analyses and microsatellite genotyping (see details in [24, 26]). The age of individuals (i.e., adult *vs*. juvenile stage) was assessed according to the body mass following Granjon & Duplantier [45] and signs of sexual maturity (i.e., external testicles and/or active seminal vesicles in males; developed mammae and uterus, presence of embryos and/or embryo scars in females). Among other samples, ethanol-preserved or directly frozen hearts and brains were collected on each small mammal specimen for subsequent molecular processing.

### Molecular detection of *T. gondii*

*Toxoplasma gondii* presence was investigated on both heart and brain samples of each small mammal collected. To do so, brains were ground separately in 1 mL of physiological water (0.9% NaCl) and 30 mg of heart cut at the apex for DNA extraction and qPCR. We relied on DNA extraction and qPCR-based screening as previously described [5, 40]. Briefly, DNA was extracted using a commercial kit as recommended by the manufacturer (Qiagen QIAamp DNA Mini Kit, Courtaboeuf, France) from 200 μL of liquid brain homogenate, and from 30 mg of the heart apex. Real-time PCR targeting the non-coding 529 bp repeat region was used to assess the presence of *T. gondii* DNA as described by Ajzenberg *et al*. [5]. Each PCR contained 5 μL of extracted DNA, mixed with 15 μL of a PCR reagent mix composed of 1X LightCycler FastStart DNA Master Hybridisation Probes kit (Roche Diagnostics, Mannheim, Germany), 0.5 U of UNG (Roche Diagnostics, Mannheim, Germany), 5 mMol/L of MgCl_2_, 0.5 μMol/L of each primer, and 0.1 μMol/L of TaqMan probe (Eurofins, Ebersberg, Germany) which is labeled with a fluorescent dye (6-carboxyfluorescein, 6-FAM) at the 5′ end and a dark quencher (Black Hole Quencher, BHQ1) at the 3′ end. A cycling protocol was run on a Rotor-Gene 6000 thermocycler (Corbett Life Science, Sydney, NSW, Australia) as follows: initial decontamination by UNG at 50 °C/2 min and denaturation at 95 °C/10 min, followed by 50 cycles at 95 °C/20 s and 60 °C/40 s. The results obtained were expressed in cycle threshold (Ct) values. Each amplification curve was visually checked to verify its sigmoidal appearance in order to avoid the inclusion of false positives. Genomic DNA isolated from Type I (RH strain, http://toxodb.org) *T. gondii* isolate cultures and sterile water were used as positive and negative qPCR controls, respectively. Each heart and brain extraction sample was investigated twice by qPCR, and individuals that provided at least one positive test were considered positive.

### Microsatellite genotyping

Genotyping relied on 15 microsatellite (MS) marker multiplex PCR corresponding to 15 loci located on 11 different *T. gondii* chromosomes [4]. Some of them were described as poorly polymorphic but still valuable for strain typing (TUB2, W35, TgM-A, B18, B17, M33, IV.1, and XI.1), while others are highly polymorphic and allow us to distinguish between strains of a same type (M48, M102, N60, N82, AA, N6, and N83) [4, 57]. The Beninese multi-locus genotypes (MLGs) were compared to reference strains described in previous studies (see Table 1) in order to reach strain-specific assignment. For genotyping, DNA isolated from Type II (ME49 strain, http://toxodb.org) *T. gondii* isolates cultures and sterile water were used as positive and negative controls, respectively.

**Table 1:** Multilocus microsatellite (MS) genotyping of micromammal-borne *Toxoplasma gondii* from Cotonou.

Isolate ID	Host species	Origin	MS alleles															Genotype	Reference
			TUB2	W35	TgM-A	B18	B17	M33	M IV.1	M XI.1	M48	M102	N60	N82	AA	N61	N83		
#803 ^ф^	*R. rattus*	APC	291	248	205	NA	342	165	274	354	229	166	NA	NA	269	NA	NA	*Africa 1*	This study
#918 ^ф; ϴ^	*C. olivieri*	Agla	291	248	205	160	342	NA	274	354	227	166	147	111	269	NA	306	*Africa 1*	This study
#954 ^ϴ^	*C. olivieri*	Agla	291	248	205	160	342	NA	274	354	221	166	149	NA	275	89	306	*Africa 1*	This study
#1000 ^ϴ^	*C. olivieri*	Ladji	291	248	205	160	342	NA	274	354	227	166	147	111	269	89	306	*Africa 1*	This study
#1491 ^ф^	*C. olivieri*	Ladji	291	248	205	NA	342	NA	274	354	225	166	147	NA	267	91	306	*Africa 1*	This study
#1589 ^ф; ϴ^	*C. olivieri*	St-Jean	291	248	205	160	342	NA	NA	354	227	NA	147	NA	267	89	NA	*Africa 1*	This study
#1616 ^ф; ϴ^	*C. olivieri*	St-Jean	291	248	NA	160	342	NA	274	354	225	NA	147	NA	273	87	306	*Africa 1*	This study
P19S1AJ6	Chicken	Benin	291	248	205	160	342	165	274	354	227	166	147	111	267	89	306	*Africa 1*	[47]
TgH 13002	Human	Senegal*	289	248	205	160	336	165	274	354	225	166	145	111	273	89	306	*Africa 2*	[57]
GAB3DOM9	Chicken	Gabon	291	242	207	160	342	165	274	354	229	166	142	111	273	95	310	*Africa 3*	[57]
P676	Chicken	Benin	293	242	203	156	336	165	274	354	215	174	129	109	291	107	306	*Africa 4*	[47]
GT1	Human	USA*	291	248	209	160	342	169	274	358	209	168	145	119	265	87	308	*Type I*	[57]
PRU	Human	France*	289	242	207	158	336	169	274	356	209	176	142	117	265	127	310	*Type II*	[57]
NED	Human	France*	289	242	205	160	336	165	278	356	209	190	147	111	267	91	312	*Type III*	[57]

*: Strains obtained from Biological Resource Center for *Toxoplasma* (http://www.toxocrb.com). Numbers indicate the length (bp) of amplified fragments at each microsatellite locus, NA: Microsatellite allele not determined at this locus. ^ф^ and ^ϴ^ Strain isolated from brain and heart, respectively.

### Statistical analyses

We used Chi-square tests on the whole dataset to compare *T. gondii* prevalence between host species, sampling localities (Agla, Ladji, Saint-Jean, and APC), and screened organs. Then, analyses of APC data (hereafter referred to as “APC-based analysis”) were performed separately from the three urban districts (hereafter referred to as “District-based analysis”) since *(i)* they display distinct socio-economic, historical and environmental characteristics, *(ii)* no landscape/GIS data were available for the APC, and *(iii)* trapping campaigns were not performed at the same period in both types of area. After determining prevalence levels of *T. gondii* (i.e., the percentage of infected hosts calculated within a 95% confidence interval (CI) estimated with Wald’s method) for each small mammal population, we carried out a three-way analysis using specific packages in RStudio v.4.1.2 software [79].

We first used Chi-square and Fisher’s exact tests to investigate possible relationships between *T. gondii* infection and certain host individual variables (sex, age, and presence of ectoparasitic fleas), period of capture (trapping session) and organs screened for *T. gondii* detection (heart or brain).

Second, we carried out generalized linear mixed models (GLMMs) on specific datasets to test whether *T. gondii* prevalence in small mammals was significantly associated with specific intrinsic and extrinsic parameters, namely the relative abundance of each species within the small mammal community (estimated by species-specific trapping success at each sampling site), host individual characteristics (sex, age, and presence/absence of fleas), period of capture (trapping session) as well as environmental features (defined by trapping site coordinates on the first four axes of PCA carried out on set landscape metrics). For the latter parameters, we took advantage of a previous study relying on the same experimental design (i.e., same sampling campaigns, hence same sampling sites) for which the dataset encompassed our own samples in the same districts of Cotonou ([27]). In this study, a wide range of landscape descriptors including landcover data, social uses associated with buildings, as well as surface water occurrences were mapped and quantified using a dedicated Geographic Information System [27]. These landscape data were integrated and explored through a principal component analysis (PCA; see Supplementary figure and Dossou *et al*. [27]). For the purpose of our study, site-specific PCA coordinates along the four first components (representing 56% of total inertia of all axes) were extracted and used as explanatory socio-environmental variables in GLMMs (only for “district-based analysis” given that this information was not collected for APC). Using the Akaike Information Criterion (AICc), we carried out a model selection procedure from a full starting model containing all predictors. *Toxoplasma gondii* prevalence was treated as a binary response (therefore assuming a binomial distribution) and either districts (for “district-based analysis”) or APC sites (for “APC-based analysis”) were considered random factors to account for possible spatial variation. Models with all possible combinations of the terms included in the starting model were generated, and the most parsimonious model (i.e., the one explaining the highest variance level with the fewest explanatory variables) was chosen among those selected within two AIC units of the best model retrieved [12]. The significance of explanatory variables and their interactions was determined by deletion testing and log-likelihood ratio tests. The final model was validated by the graphical checking of normality, independence, and variance homogeneity of residuals.

Lastly, as implemented in Mariën *et al.* [55] and Sirdar *et al.* [76], possible spatial/site clustering of *T. gondii*-carrying reservoirs was investigated in the sampling localities through Cuzick–Edwards (nearest-neighbour) tests [19, 70]. To do so, Monte-Carlo permutations (*n* = 10,000) were run to infer whether clustering of the field data was significantly higher (*p* ≤ 0.05) than under random simulations [36]. After confirming that *T. gondii*-positive animals were significantly clustered, we used Kulldorff’s spatial scan statistics in order to characterize the local clusters, which make it possible to assess the number and geographical position of local clusters, as well as the number of rodents involved in the potential clusters identified [53].

All these analyses were performed using the following packages: lme4 [9] for GLMMs, MuMIN [8] for model selection, Ade4 for PCA [28], and Smacpod [36] and Spatstat [7] for spatial clustering analysis.

## Results

### Sampling

We analyzed a total of 632 small mammals belonging to seven species: Black rat, *Rattus rattus* (*n* = 234 individuals), African giant shrew, *Crocidura olivieri* (*n* = 179), House mouse, *Mus musculus domesticus* (*n* = 102), Brown rat, *Rattus norvegicus* (*n* = 77), Natal multimammate mouse, *Mastomys natalensis* (*n* = 28), Gambian pouched rat, *Cricetomys gambianus* (*n* = 7) and Deroo’s mouse, *Praomys derooi* (*n* = 5). These host populations comprised 481 adults and 106 juveniles (the age of 45 individuals could not be unambiguously determined) and 282 males and 350 females. Only 13.5% (81 out of 598) of individuals carried at least one flea. In our dataset, *R. rattus* (Agla: *n* = 45; Ladji: *n* = 58; Saint-Jean: *n* = 34; and APC: *n* = 97) as well as *C. olivieri* (Agla: *n* = 39; Ladji: *n* = 49; Saint-Jean: *n* = 34; and APC: *n* = 57) showed high levels of presence in all localities. *Mastomys natalensis* was mostly trapped in the districts (97.5% of all *Mastomys* individuals captured), while *R. norvegicus* and *M. m. domesticus* were mostly trapped in APC with 79.2% and 100% of individuals captured in this locality, respectively (see Fig. 1 and Supplementary Tables 1 & 2 for details).

### Molecular prevalence of *T. gondii*

#### Overall localities in Cotonou

*Toxoplasma* DNA was detected in 96 small mammals, thus representing an overall molecular prevalence of 15.2% (95% [CI = 12.39; 17.99]) (see Supplementary Tables 1 & 2 for details of prevalence). Regarding localities, the highest prevalence was found in Saint-Jean and APC, with 18.4% [10.25; 26.53] and 17.1% [12.93; 21.13], respectively, followed by Ladji with 12.2% [6.20; 18.15] and Agla with 10.3% [4.53; 16.03]. However, Chi2 tests revealed that differences were not significant between localities (χ^2^ = 4.4; *p* = 0.23). Regarding the host species, the highest prevalence was found in *M. m. domesticus* and *C. olivieri* with 20.6% [12.74; 28.44] and 19.6% [13.74; 25.36], respectively, followed by *M. natalensis* with 14.3% [0.0; 40.21], *R. norvegicus* with 14.3% [6.47; 22.10] and *R. rattus* with 9.9% [6.01; 13.64]. The two species *C. gambianus* and *P. derooi*, which were poorly represented in the sampling (*n* = 7 and *n* = 5), had only one and two individuals infected, respectively and were not taken into account in the subsequent analyses. Regarding the targeted organ, no significant differences were found between the brain and heart, with prevalence reaching 9.9% [7.63; 12.3] and 7.4% [5.25; 9.3], respectively. Only thirteen individuals displayed positive results in both organs (13.5% [9.49; 17.56]).

#### District-based analysis

For the three sampled districts, comparison of the prevalence showed a significant difference between species (χ^2^ = 9.9; *p* = 0.02), with pairwise comparisons indicating that shrews were significantly more infected than black rats (χ^2^ = 8.8; *p* = 0.003). No statistical pairwise difference was found between other species. Regarding the district sampled, no significant difference was found (χ^2^ = 2.9; *p* = 0.23). Regarding the trapping session, no difference was found between species nor between districts, except in Saint-Jean where prevalence was higher in June 2018 than in October 2017 (χ^2^ = 8.2; *p* = 0.004). Finally, regardless of the species considered, there was no significant relationship between *T. gondii* infection and respectively sex, age, or flea carriage (χ^2^, all *p* > 0.2).

#### APC-based analysis

Our analyses excluded *M. natalensis*, for which only six individuals were trapped and none of them were infected. We found no significant difference in *T. gondii* infection between other species (χ^2^ = 1.8; *p* = 0.62). *Toxoplasma gondii* prevalences in the mouse population were significantly higher in September–November 2017 than in March 2018 (χ^2^ = 5.6; *p* = 0.02), while no differences were found in other species depending on the trapping period. Irrespective of the species considered, there was no sex, age, and fleas carriage-associated distribution of *T. gondii* infection (χ^2^, all *p* > 0.3) except in *R. norvegicus* where juveniles seemed to be more commonly infected than adults (Fisher’s test, *p* = 0.005).

### Genotyping

Most of the qPCR-positive small mammals displayed DNA concentrations of *T. gondii* in their tissues that were too low to enable genotyping (Ct > 32). Samples from seven individuals (samples #803, 918, 954, 1000, 1491, 1589 and 1616) had sufficient *T. gondii* DNA for partially successful MS-markers amplification (10/15 to 14/15 amplified markers). Despite incomplete genotyping, enough markers were amplified for lineage identification: all seven samples belonged to the *Africa 1* lineage genotypes (Table 1). These genotypes were retrieved from one black rat captured in the APC and two shrews in each of the three urban neighbourhoods.

### Influence of intrinsic/extrinsic factors on *T. gondii* molecular prevalence

Several GLMMs were tested, either considering all small mammals species investigated or separately for each species with at least 50 individuals sampled, in both “district-based” and “APC-based” models (Table 2).

**Table 2:** Results of Generalized Linear Mixed Models (best models) explaining *T. gondii* infection of main rodent host species in the sampled districts. Rra, Rno, Mna, Cro, Mus represent *Rattus rattus*, *Rattus norvegicus, Mastomys natalensis, Crocidura olivieri and Mus musculus domesticus* yield, respectively; Fleas: presence of fleas on the individual. Session: trapping session. PCA 1–4: PCA sites coordinates of axis 1–4.

Localities	Species^a^	Explanatory variables	*N* ^b^	Predictors^c^	Estimate ± SE	*p*-value
Districts (Agla, Ladji & Saint-Jean)	All species	Species, Sex, Age, Fleas, Session, PCA1, PCA2, PCA3, PCA4, Rra, Rno, Mna, Cro	260	Rra	−6.3668 ± 2.6852	0.004
				Cro	5.9944 ± 2.6073	0.02
	*C. olivieri*	Sex, Age, Fleas, Session, PCA1, PCA2, PCA3, PCA4, Rra, Rno, Mna	91	None	–	–
	*R. rattus*	Sex, Age, Fleas, Session, PCA1, PCA2, PCA3, PCA4, Rno, Mna, Cro	125	None	–	–
APC	All	Species, Sex, Age, Fleas, Session, Rra, Rno, Mna, Cro, Mus	295	Mus	7.256 ± 2.663	0.007
	*C. olivieri*	Sex, Age, Fleas, Session, Rra, Rno, Mna, Mus	50	None	–	–
	*R. rattus*	Sex, Age, Fleas, Session, Rno, Mna, Cro, Mus	89	None	–	–
	*R. norvegicus*	Sex, Age, Fleas, Session, Rra, Mna, Cro, Mus	54	None	–	–
	*M. m. domesticus*	Sex, Age, Fleas, Session, Rra, Rno, Mna, Cro	96	Session	1.6045 ± 0.6653	0.006

a Species included in the model.

b Total number of individuals included in model selection.

c Selected variables.

#### District-based GLMMs

We found a parsimonious model only when considering all small mammal species. A high *T. gondii* prevalence was *(i)* negatively associated with *R. rattus* relative abundance (χ^2^ = 7.99; *p* = 0.005), and *(ii)* positively associated with *C. olivieri* relative abundance (χ^2^ = 5.06; *p* = 0.024). Therefore, the socio-environmental proxies used this study failed to show any influence on *T. gondii* prevalence in small mammal communities.

#### APC-based GLMMs

Considering all small mammal species, *T. gondii* infection was positively correlated with the relative abundance of *M. m. domesticus* (χ^2^ = 7.22; *p* = 0.007). For this rodent species, the most parsimonious model best explaining *T. gondii* infection included the trapping session (χ^2^ = 7.45; *p* = 0.006) only; they were significantly more commonly infected in September–November 2017 than in March 2018.

### Spatial clustering of *T. gondii-*infected small mammals

**Figure 2:**
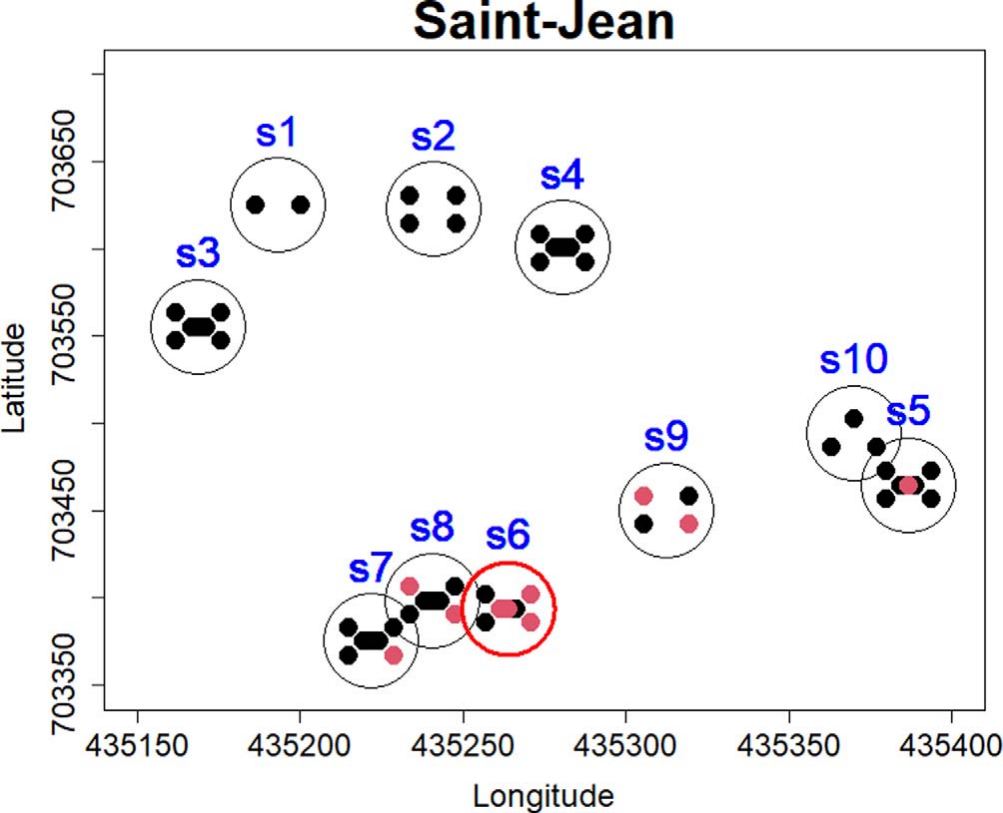
Spatial clustering of *T. gondii*-infected small mammals of Cotonou. Each black circle corresponds to a sampled site graphically represented by its GPS coordinates. Red and black dots represent *T. gondii*-infected and *T. gondii*-uninfected animals, respectively. Red circles represent significant clusters of cases based on the spatial statistic scan.

Regardless of the trapping period considered, no significant clusters were observed in the four studied localities; however, when the two trapping sessions were grouped, a significant clustering of *T. gondii-*infected animals was observed in Saint-Jean district, with Monte Carlo *p-*values lower than 0.05 for *q* = 10 nearest neighbours (*p-*value = 0.03) based on the Cuzick-Edwards test (Table 3). Kulldorff’s spatial statistic scan also indicated a single significant local cluster (*p-*value = 0.005) at one given Saint-Jean site (i.e., S6) where 5 out of 9 individuals were infected (Fig. 2).

**Table 3:** Monte Carlo *p*-values (10,000 simulations) to investigate significant clustering of *T. gondii*-infected small mammals within each study area based on the Cuzick–Edwards test.

Localities	Session	Total^a^	N. Pos^b^	*N*^c^ = 2	*N* = 4	*N* = 6	*N* = 8	*N* = 10	Kulldorf *p*-values
Agla	Sept/Nov 2017	50	6	0.37	0.68	0.86	0.49	0.17	0.36
	June 2018	57	5	0.4	0.46	0.19	0.16	0.08	0.14
	All	107	11	0.22	0.55	0.66	0.53	0.16	0.02
Ladji	Sept/Nov 2017	52	9	0.52	0.48	0.82	0.25	0.31	0.18
	June 2018	63	5	0.37	0.61	0.58	0.41	0.2	0.78
	All	115	14	0.16	0.10	0.07	0.24	0.33	0.02
St-Jean	Sept/Nov 2017	46	3	0.2	0.16	0.26	0.06	0.07	0.07
	June 2018	40	13	0.07	0.32	0.08	0.07	0.09	0.2
	All	86	16	0.72	0.63	0.14	0.1	**0.03**	**0.005**
PAC	Oct 2017	162	18	0.94	0.87	0.9	0.89	0.91	0.42
	March 2018	159	16	0.67	0.64	0.61	0.77	0.74	0.84
	All	321	54	0.8	0.64	0.57	0.58	0.61	0.12

a Total number of captured rodents.

b Total number of *T. gondii*-Infected rodents.

c Number of nearest neighbours (*q*).

Bold numbers were considered to be significant.

## Discussion

To our knowledge, the present study is the first to focus on interrelationships between several abiotic and rodent-based biotic factors and *T. gondii* circulation in African urban dwellings. Our results are consistent with previous findings from Europe and North America, showing that small mammals play an important role in the maintenance and circulation of *T. gondii* within the urban environment [11, 30, 40–42]. Furthermore, our qPCR-based approach led to higher prevalence values (20.6% in *M. m. domesticus*, 19.6% in *C. olivieri,* 14.3% in *M. natalensis*, 14.3% in *R. norvegicus* and 9.9% in *R. rattus*) than those previously observed elsewhere using molecular methods, regardless of the *T. gondii* targeted gene, the methodology of screening, and the geographical scale considered (e.g., in *R. rattus*: 1.8% in São Paolo, Brazil [60]; 4.5% in Gran La Plata, Argentina [23]; 4.3% in Sichuan, China [85]). In the same manner, within the African context, the prevalence exhibited here was also higher than that found in Senegal with the same qPCR-based approach (i.e., 3.8% in black rats, 13.3% in house mice, and 15.5% in shrews; [40]). These differences in prevalence could result from the nature of the tissues analyzed. Importantly, the study in Senegal (and in most studies) was based only on brain samples, whereas our study relied on both brain and heart. Although no significant differences in molecular prevalence were found between organs, only 13.5% of the 96 positive animals displayed positive results in both organs. When focusing only on brain samples, our results show a prevalence of 6.4% in black rats, 14.7% in house mice, and 14.5% in shrews, which is much closer to the prevalence observed in Senegal [40]. This suggests that both organs (brain and heart) should be considered together when investigating molecular prevalence of *T. gondii* in future small mammal-centered surveys. Another non-exclusive hypothesis on the differences observed between the Senegalese and Beninese contexts could be the differences in socio-environmental conditions (e.g., climate, nature of soil, vegetation type) between both countries. As such, sampled localities in Senegal (Dakar, Rufisque and Joal-Fadiouth [40]) are characterized by a hot semi-arid climate [33], which could contribute to reduce the survival rate of *T. gondii* oocysts, while Cotonou is favorably more humid, with recurring floods [52, 86]. For a clearer view, we advocate for study of different geographic contexts of *T. gondii* epidemiology within commensal small mammal communities showing contrasted eco-evolutionary trajectories.

Nevertheless, our analyses provide interesting insights on the tested factors. As previously described by Galal *et al*. [40], we showed that shrews were significantly more often infected than black rats over the three Cotonou districts. This could be due to differences in life traits: black rats are omnivorous and good climbers, while shrews forage only on the ground where they show marked digging and exploratory behavior for feeding [10, 15, 16]. The latter habits may likely favor contact with *T. gondii* oocysts excreted in the environment by stray and/or domestic cats. Moreover, no gender difference was observed for *T. gondii* infection for any of the reservoir species investigated here. This was congruent with findings outlined elsewhere [20, 58, 67], suggesting that gender-based differences in rodent behavior, immunity, and/or physiology do not influence *T. gondii* infection. Within-species comparisons also showed no difference in infection with age, except in APC where juveniles were more commonly infected than adults in *R. norvegicus*. Nonetheless, we should be cautious when interpreting this outcome given the low number of juveniles trapped for certain species, including *R. norvegicus* (e.g., in APC: 7 juveniles out of 61 individuals). Regardless of the locality and the small mammal species, no relationship was found between *T. gondii* prevalence and flea carriage. Although ectoparasites have been hypothesized as paratenic hosts and vectors of *T. gondii* [66, 77, 87], our results did not allow us to identify a significant relationship between *T. gondii* infection and the presence of ectoparasitic fleas, suggesting that flea parasitism does not appear to influence *T. gondii* infection in small mammals.

Furthermore, except for Saint-Jean where prevalence was higher in June 2018 than in October 2017 (32.5% *vs.* 6.4%), no differences in *T. gondii* prevalence were found between trapping sessions irrespective of the district or the species considered. This observation was also made for rodent-borne *T. gondii* seroprevalence in Niamey city (Niger) [58], suggesting that infection in small mammals may consistently occur throughout the year in West African urban regions. Nevertheless, we cannot exclude that the difference observed in Saint-Jean may be because both trapping sessions correspond to the rainy season. However, our data are restricted to only two trapping sessions, thus precluding any robust and definitive conclusion about potential seasonal variations in small mammal-borne *T. gondii* prevalence. Temporal surveys during each season and over at least two years would be required to capture such possible temporal dynamics*.*

The “district-based GLMMs” for explaining *T. gondii* infections did not show any clear influence of the local socio-environment – at least as defined by the proxies used in this study. This finding may be interpreted in the light of the ubiquitous nature of *T. gondii*, which is adapted to diverse environmental matrices, including soil, vegetation, standing water, and the marine environment [71, 86] – and even in polar areas where felids are absent [31]. Rather than being impacted by socio-environmental determinants, *T. gondii* prevalence in Cotonou was shown to be related to the relative abundances of black rats and shrews. In fact, a high prevalence of the parasite appeared to be significantly associated with a low abundance of *R. rattus*, and a high abundance of *C. olivieri*. The social and feeding behavior of *R. rattus* could explain the low prevalence of *T. gondii* observed in this species. As explained above, the black rat is a good climber which often builds nests in roofs of houses [34], which might limit direct contact with the bare soil where *T. gondii* oocysts disseminate. Moreover, the large size of *Rattus* species [18, 64] makes them less prone to predation by domestic cats which act as final hosts [14, 35, 65, 78]. However, this interpretation, outlined elsewhere [1, 40, 67], should be taken with caution since the main mode of *T. gondii* transmission in natural small mammal populations is still unknown [32]. Alternatively, shrews may appear to be an important host for the circulation of *T. gondii* within the urban environment. Altogether, it appears that species-specific susceptibility to *Toxoplasma* may be a key element of the ecology of this ubiquitous parasite. Therefore, we stress the need to further investigate the intricacies of host-*T. gondii* interactions at the species level.

Our “APC-based GLMMs” showed that the probability of *T. gondii* infection in the small mammal community was positively correlated with the abundance of *M. m. domesticus*. This rodent species is generally considered to be the preferred prey of domestic cats [35, 65], which could lead to increasing rates of oocysts excretion in the environment and thus higher infection opportunities for other species, including small mammals. Of note, although probably not very frequent, cats were sometimes observed wandering inside the seaport storehouses during our field trapping campaigns. Moreover, in wild-derived mice, the hypothesis of the maintenance of *T. gondii* infection over several generations by congenital transmission has been formulated [50, 56, 62, 63], and might at least partly explain the high prevalence observed in APC where all house mice were caught. Mice were found to be significantly more infected in September 2017 (rainy season) than in March 2018 (end of the dry season). Assuming environmental contamination, such a trend could be explained by the characteristics of sampled APC sites. Mice were essentially trapped in the industrial zones of APC that are characterized by large cemented surfaces and a total absence of vegetation. These conditions could be particularly unfavorable to the survival of oocysts during the dry season. Such a hypothesis is congruent with wet weather and water runoff as factors facilitating *T. gondii* oocyst survival and/or dissemination [67, 80, 84], and might explain the high prevalence observed in APC mice during the rainy season. From there, spatio-temporal surveys over different seasons in this very peculiar environment are needed to draw robust conclusions.

No significant spatial cluster of *T. gondii*-infected animals was observed in Agla, Ladji and APC, but one significant cluster was retrieved in Saint-Jean – at site S6 where 56% of individuals were found to be infected. This site is adjacent to sites S7 and S8 where prevalence reached 27% (4/15) and 30% (3/10), respectively. This cluster can be interpreted as a local hotspot of parasite circulation. Based on our knowledge of the major routes of *T. gondii* transmission to intermediate hosts, we would expect the parasite infection to be strongly dependent on cat density [3, 29, 43, 44, 88]. Unfortunately, to our knowledge, robust data on cat abundances and activities in Cotonou are not available. During a previous survey (Houémènou *et al*., personal information) in the exact same households of Agla, Ladji and Saint-Jean conducted from September 2016 to June 2017 (i.e., just before the start of the sampling campaigns for the present study in October 2017 and June 2018), cats were observed or indicated by inhabitants at least once in 2/10 sites in Ladji, 3/10 sites in Agla, and up to 5/10 sites in Saint-Jean, thus allowing a rough indication of cat abundance in our districts. The apparently frequent occurrence of domestic cats in Saint-Jean households could explain the local hotspot identified by our clustering analyses. However, in the absence of reliable data, this hypothesis remains to be formally tested and it would therefore be critical in future studies on *Toxoplasma* eco-epidemiology in southern Benin to take cats (and other potentially impacting factors, such as climate data, co-infection, and human exposure) into account, since they should represent rarely investigated but obviously crucial factors.

The seven samples genotyped here were all classified as belonging to the *Africa 1* lineage, a *T. gondii* lineage widespread in most West and Central African countries, including Benin [38, 40, 47, 57]. They were retrieved in six shrews (two individuals in each urban district) and one black rat (originating from APC). Although the number of genotyped individuals is low, it nonetheless confirms the predominance of *Africa 1* strain in domestic and peri-domestic environments of Southern Benin, including Cotonou, as previously shown in chickens [47] and, beyond, throughout tropical Africa on different hosts [38, 57]. The *Africa 1* strain is considered lethal in laboratory and wild mice (*Mus musculus domesticus*) [40, 47, 49, 57] and the link between strain virulence and host availability in shaping genetic diversity of *Toxoplasma* worldwide is supported by an increasing number of studies [37, 54, 72]. In Senegal, the invasive house mouse *Mus musculus domesticus* is currently the most abundant rodent in seaport cities such as Dakar and Saint-Louis, as well as in several central and northern localities where most *T. gondii* isolates belong to non-mouse virulent lineages [21]. On the contrary, this invasive rodent species has not yet disseminated to certain inland regions where the *Africa 1* mouse-virulent *T. gondii* lineage was shown to be predominant [39]. In Benin, house mice invasion is probably still in its earliest phases since this species has been described almost only within APC [25, 51]. These differences in the respective history and dynamics of the house mouse invasion taking place in these two countries may explain the distinct *T. gondii* strains that circulate among local *M. musculus* populations. Testing such a hypothesis requires us to investigate the virulence of the autochthonous *Africa 1* strain in invasive house mice from Senegal and Benin. Extended genomic analyses to better capture and understand the diversity of circulating *T. gondii* strains from wild small mammal are needed since infectivity and morbidity of toxoplasmosis was shown to be related to the protozoan genotype involved [48, 72, 75].

In conclusion, our study provides a new support for the ecological ubiquity of *T. gondii* in a West-African country. Especially, we *(i)* showed the need to investigate both brain and heart organs in molecular surveys of *T. gondii* to draw a more accurate picture of the parasite prevalence among small mammals, *(ii)* confirmed the dominance of *Africa 1* lineage as the dominant strain within West-African urban areas, and *(iii)* highlighted the intricate interrelationships between several biotic and abiotic determinants that make the circulation of *T. gondii* a local context-dependent process in which the underlying factors seem to differ – even at a very local scale – from one socio-environmental situation to another. From all the foregoing, multidisciplinary studies are key to integrate socio-economic, biological and environmental components when studying the ecology and transmission to humans of *T. gondii* in domestic environments.

## Funding

This work was supported by funds from the French *Agence Nationale de la Recherche* (ANR project IntroTox 17-CE35-0004 given to A. Mercier), the Nouvelle Aquitaine region of France, and recurrent funding (given to G. Dobigny) from the French Institute of Research for Sustainable Development (IRD). The funders had no role in study design, data collection and analysis, decision to publish, or preparation of the manuscript.

## Authors contributions

Design of the study: A. Mercier and G. Dobigny; Sampling: J. R. Etougbétché, H.-J. Dossou, S. Badou, and G. Dobigny; Molecular biology work: A. Hamidović, N. Plault, M. Coan-Grosso, R. Roques; Data analysis and processing: J. R. Etougbétché, C. Diagne, H.-J. Dossou; Paper writing & review: J. Etougbétché, A. Hamidović, Lokman Galal, C. Diagne, G. Dobigny, A. Mercier. Supervision: A. Mercier, G. Dobigny, M-L Dardé, G. Houéménou, A. A. Missihoun and I. Youssao Abdou Karim.

## Supplementary material

The Supplementary material of this article is available at https://www.parasite-journal.org/10.1051/parasite/2022058/olm. *Supplementary Table 1*: Captures and species-specific prevalence by sampled localities and sites *Supplementary Table 2*: Prevalence by sex, age, session, and flea carriage. *Supplementary Figure*: Biplot PCA Cotonou, axis 1 & 2 and 3 & 4.

## References

[1] Afonso E, Poulle M-L, Lemoine M, Villena I, Aubert D, Gilot-Fromont E. 2007. Prevalence of *Toxoplasma gondii* in small mammals from the Ardennes region, France. Folia Parasitologica, 54, 313. 18303774 10.14411/fp.2007.041

[2] Afonso E, Thulliez P, Pontier D, Gilot-Fromont E. 2007. Toxoplasmosis in prey species and consequences for prevalence in feral cats: not all prey species are equal. Parasitology, 134, 1963–1971. 17672925 10.1017/S0031182007003320

[3] Ajmal A, Maqbool A, Qamar MF, Ashraf K, Anjum AA. 2013. Detection of *Toxoplasma gondii* in environmental matrices (water, soil, fruits and vegetables). African Journal of Microbiology Research, 7, 1505–1511.

[4] Ajzenberg D, Collinet F, Mercier A, Vignoles P, Dardé M-L. 2010. Genotyping of *Toxoplasma gondii* isolates with 15 microsatellite markers in a single multiplex PCR assay. Journal of Clinical Microbiology, 48, 4641–4645. 20881166 10.1128/JCM.01152-10 PMC3008440

[5] Ajzenberg D, Lamaury I, Demar M, Vautrin C, Cabié A, Simon S, Nicolas M, Desbois-Nogard N, Boukhari R, Riahi H. 2016. Performance testing of PCR assay in blood samples for the diagnosis of toxoplasmic encephalitis in AIDS patients from the French Departments of America and genetic diversity of *Toxoplasma gondii*: a prospective and multicentric study. PLoS Neglected Tropical Diseases, 10, e0004790. 27355620 10.1371/journal.pntd.0004790 PMC4927177

[6] Antoniou M, Psaroulaki A, Toumazos P, Mazeris A, Ioannou I, Papaprodromou M, Georgiou K, Hristofi N, Patsias A, Loucaides F. 2010. Rats as indicators of the presence and dispersal of pathogens in Cyprus: ectoparasites, parasitic helminths, enteric bacteria, and encephalomyocarditis virus. Vector-Borne and Zoonotic Diseases, 10, 867–873. 20370433 10.1089/vbz.2009.0123

[7] Baddeley A, Turner R. 2005. Spatstat: an R package for analyzing spatial point patterns. Journal of Statistical Software, 12, 1–42.

[8] Barton K, Barton MK. 2015. Package “mumin.” Version, 1, 439.

[9] Bates D, Mächler M, Bolker B, Walker S. 2015. Fitting linear mixed-effects models using lme4. Journal of Statistical Software, 67(1), 1–48. https://doi.org/10.18637/jss.v067.i01.

[10] Brahmi K, Aulagnier S, Slimani S, Mann CS, Doumandji S, Baziz B. 2012. Diet of the greater white-toothed shrew *Crocidura russula* (Mammalia: Soricidae) in Grande Kabylie (Algeria). Italian Journal of Zoology, 79, 239–245.

[11] Brouat C, Diagne CA, Ismaïl K, Aroussi A, Dalecky A, Bâ K, Kane M, Niang Y, Diallo M, Sow A, Galal L, Piry S, Dardé M-L, Mercier A. 2018. Seroprevalence of *Toxoplasma gondii* in commensal rodents sampled across Senegal, West Africa. Parasite, 25, 32. 30016257 10.1051/parasite/2018036 PMC6050035

[12] Burnham KP, Anderson DR. 2002. A practical information-theoretic approach. Model Selection and Multimodel Inference, 2, 70–71.

[13] Castel G, Kant R, Badou S, Etougbétché J, Dossou H-J, Gauthier P, Houéménou G, Smura T, Sironen T, Dobigny G. 2021. Genetic characterization of Seoul virus in the seaport of Cotonou, Benin. Emerging Infectious Diseases, 27, 2704. 34545795 10.3201/eid2710.210268 PMC8462318

[14] Childs JE. 1986. Size-dependent predation on rats (*Rattus norvegicus*) by house cats (*Felis catus*) in an urban setting. Journal of Mammalogy, 67, 196–199.

[15] Churchfield S, Barrière P, Hutterer R, Colyn M. 2004. First results on the feeding ecology of sympatric shrews (Insectivora: Soricidae) in the Tai National Park, Ivory Coast. Acta Theriologica, 49, 1–15.

[16] Clausnitzer V, Churchfield S, Hutterer R. 2003. Habitat occurrence and feeding ecology of *Crocidura montis* and *Lophuromys flavopunctatus* on Mt. Elgon, Uganda. African Journal of Ecology, 41, 1–8.

[17] Cola GA, Garcia JL, da Costa L, Ruffolo B, Navarro IT, Freire RL. 2010. Comparison of the indirect fluorescent antibody test and modified agglutination test for detection of anti-*Toxoplasma gondii* antibodies in rats. Semina: Ciências Agrárias, 31, 717–722.

[18] Combs M, Puckett EE, Richardson J, Mims D, Munshi-South J. 2018. Spatial population genomics of the brown rat (*Rattus norvegicus*) in New York City. Molecular Ecology, 27, 83–98. 29165929 10.1111/mec.14437

[19] Cuzick J, Edwards R. 1990. Spatial clustering for inhomogeneous populations. Journal of the Royal Statistical Society: Series B (Methodological), 52, 73–96.

[20] Dabritz HA, Miller MA, Gardner IA, Packham AE, Atwill ER, Conrad PA. 2008. Risk factors for *Toxoplasma gondii* Infection in wild rodents from Central Coastal California and a review of *T. gondii* prevalence in rodents. Journal of Parasitology, 94, 675–683. 18605783 10.1645/GE-1342.1

[21] Dalecky A, Bâ K, Piry S, Lippens C, Diagne CA, Kane M, Sow A, Diallo M, Niang Y, Konečnỳ A. 2015. Range expansion of the invasive house mouse *Mus musculus domesticus* in Senegal, West Africa: a synthesis of trapping data over three decades, 1983–2014. Mammal Review, 45, 176–190.

[22] Dardé ML. 2008. *Toxoplasma gondii*, “new” genotypes and virulence. Parasite, 15, 366–371. 18814708 10.1051/parasite/2008153366

[23] Dellarupe A, Fitte B, Pardini L, Campero LM, Bernstein M, Robles M del R, Moré G, Venturini MC, Unzaga JM. 2019. *Toxoplasma gondii* and *Neospora caninum* infections in synanthropic rodents from Argentina. Revista Brasileira de Parasitologia Veterinária, 28, 113–118. 30916257 10.1590/S1984-29612019009

[24] Dobigny G, Gauthier P, Houéménou G, Dossou HJ, Badou S, Etougbétché J, Tatard C, Truc P. 2019. Spatio-temporal survey of small mammal-borne *Trypanosoma lewisi* in Cotonou, Benin, and the potential risk of human infection. Infection, Genetics and Evolution, 75, 103967. 10.1016/j.meegid.2019.103967 31344489

[25] Dossou H-J, Adjovi N, Houéménou G, Bagan T, Mensah G-A, Dobigny G. 2020. Invasive rodents and damages to food stocks: a study in the Autonomous Harbor of Cotonou, Benin. Biotechnologie, Agronomie, Société et Environnement/Biotechnology. Agronomy, Society and Environment, 24, 28–36.

[26] Dossou H-J, Le Guyader M, Gauthier P, Badou S, Etougbetche J, Houemenou G, Djelouadji Z, Dobigny G. 2022. Fine-scale prevalence and genetic diversity of urban small mammal-borne pathogenic Leptospira in Africa: A spatiotemporal survey within Cotonou, Benin. Zoonoses and Public Health, 69, 643–654. 35524648 10.1111/zph.12953 PMC9540415

[27] Dossou H-J, Tenté B, Houémènou G, Sossou MD, Rossi J-P, Dobigny G. 2021. Fine-scale Landscape Variability of Cotonou City. Insights From Three Contrasted Urban Neighborhoods: Benin.

[28] Dray S, Dufour A-B. 2007. The ade4 package: implementing the duality diagram for ecologists. Journal of Statistical Software, 22, 1–20.

[29] Du F, Zhang Q, Yu Q, Hu M, Zhou Y, Zhao J. 2012. Soil contamination of *Toxoplasma gondii* oocysts in pig farms in central China. Veterinary Parasitology, 187, 53–56. 22265079 10.1016/j.vetpar.2011.12.036

[30] Dubey JP, Murata FHA, Cerqueira-Cézar CK, Kwok OCH, Su C. 2021. Epidemiological significance of *Toxoplasma gondii* Infections in wild rodents: 2009–2020. Journal of Parasitology, 107, 182–204. 33662119 10.1645/20-121

[31] Dubey JP, Murata FHA, Cerqueira-Cézar CK, Kwok OCH, Su C. 2021. Epidemiologic and public health significance of *Toxoplasma gondii* Infections in bears (*Ursus spp.*): a 50 year review including recent genetic evidence. Journal of Parasitology, 107, 519–528. 34167147 10.1645/21-16

[32] Dubey JP. 2009. Toxoplasmosis of Animals and Humans, 2th edition. CRC Press: Boca Raton. 10.1201/9781420092370.

[33] Faye C. 2019. Changement climatiques observés sur le littoral sénégalais (Région de Dakar) depuis 1960: Étude de la variabilité des tendances sur les températures et la pluviométrie.

[34] Feng AY, Himsworth CG. 2014. The secret life of the city rat: a review of the ecology of urban Norway and black rats (*Rattus norvegicus* and *Rattus rattus*). Urban Ecosystems, 17, 149–162.

[35] Fleming PA, Crawford HM, Auckland CH, Calver MC. 2020. Body size and bite force of stray and feral cats – Are bigger or older cats taking the largest or more difficult-to-handle prey? Animals, 10, 707. 32316555 10.3390/ani10040707 PMC7222765

[36] French J. 2018. smacpod: Statistical Methods for the Analysis of Case-Control Point Data. R Package Version, 2 p.

[37] Galal L, Hamidović A, Dardé ML, Mercier M. 2019. Diversity of *Toxoplasma gondii* strains at the global level and its determinants. Food and Waterborne Parasitology, 15, e00052. 32095622 10.1016/j.fawpar.2019.e00052 PMC7033991

[38] Galal L, Ajzenberg D, Hamidović A, Durieux M-F, Dardé M-L, Mercier A. 2018. *Toxoplasma* and Africa: One parasite, two opposite population structures. Trends in Parasitology, 34, 140–154. 29174610 10.1016/j.pt.2017.10.010

[39] Galal L, Sarr A, Cuny T, Brouat C, Coulibaly F, Sembène M, Diagne M, Diallo M, Sow A, Hamidović A, Plault N, Dardé M-L, Ajzenberg D, Mercier A. 2019. The introduction of new hosts with human trade shapes the extant distribution of *Toxoplasma gondii* lineages. PLOS Neglected Tropical Diseases, 13, e0007435. 31295245 10.1371/journal.pntd.0007435 PMC6622481

[40] Galal L, Schares G, Stragier C, Vignoles P, Brouat C, Cuny T, Dubois C, Rohart T, Glodas C, Dardé M-L, Kane M, Niang Y, Diallo M, Sow A, Aubert D, Hamidović A, Ajzenberg D, Mercier A. 2019. Diversity of *Toxoplasma gondii* strains shaped by commensal communities of small mammals. International Journal for Parasitology, 49, 267–275. 30578812 10.1016/j.ijpara.2018.11.004

[41] Galeh TM, Sarvi S, Hosseini SA, Daryani A. 2022. Genetic diversity of *Toxoplasma gondii* isolates from rodents in the world: A systematic review. Transboundary and Emerging Diseases, 69, 943–957. 33825346 10.1111/tbed.14096

[42] Galeh TM, Sarvi S, Montazeri M, Moosazadeh M, Nakhaei M, Shariatzadeh SA, Daryani A. 2020. Global Status of *Toxoplasma gondii* seroprevalence in rodents: A systematic review and meta-analysis. Frontiers in Veterinary Science, 7, 461. 32851037 10.3389/fvets.2020.00461 PMC7411222

[43] Gotteland C, Chaval Y, Villena I, Galan M, Geers R, Aubert D, Poulle M-L, Charbonnel N, Gilot-Fromont E. 2014. Species or local environment, what determines the infection of rodents by *Toxoplasma gondii*? Parasitology, 141, 259–268. 24135380 10.1017/S0031182013001522

[44] Gotteland C, Gilot-Fromont E, Aubert D, Poulle M-L, Dupuis E, Dardé M-L, Forin-Wiart M-A, Rabilloud M, Riche B, Villena I. 2014. Spatial distribution of *Toxoplasma gondii* oocysts in soil in a rural area: Influence of cats and land use. Veterinary Parasitology, 205, 629–637. 25178554 10.1016/j.vetpar.2014.08.003

[45] Granjon L, Duplantier J-M. 2009. Les rongeurs de l’Afrique sahélo-soudanienne. IRD.

[46] Grzybek M, Antolová D, Tołkacz K, Alsarraf M, Behnke-Borowczyk J, Nowicka J, Paleolog J, Biernat B, Behnke JM, Bajer A. 2021. Seroprevalence of *Toxoplasma gondii* among sylvatic rodents in Poland. Animals, 11, 1048. 33917803 10.3390/ani11041048 PMC8068096

[47] Hamidović A, Etougbétché JR, Tonouhewa ABN, Galal L, Dobigny G, Houémènou G, Da Zoclanclounon H, Amagbégnon R, Laleye A, Fievet N. 2021. A hotspot of *Toxoplasma gondii* Africa 1 lineage in Benin: How new genotypes from West Africa contribute to understand the parasite genetic diversity worldwide. PLoS Neglected Tropical Diseases, 15, e0008980. 33571262 10.1371/journal.pntd.0008980 PMC7904144

[48] Hamilton CM, Black L, Oliveira S, Burrells A, Bartley PM, Melo RPB, Chianini F, Palarea-Albaladejo J, Innes EA, Kelly PJ, Katzer F. 2019. Comparative virulence of Caribbean, Brazilian and European isolates of *Toxoplasma gondii*. Parasites & Vectors, 12, 104. 30871587 10.1186/s13071-019-3372-4 PMC6416883

[49] Hassan MA, Olijnik A-A, Frickel E-M, Saeij JP. 2019. Clonal and atypical *Toxoplasma* strain differences in virulence vary with mouse sub-species. International Journal for Parasitology, 49, 63–70. 30471286 10.1016/j.ijpara.2018.08.007 PMC6344230

[50] Hide G, Morley EK, Hughes JM, Gerwash O, Elmahaishi MS, Elmahaishi KH, Thomasson D, Wright EA, Williams RH, Murphy RG. 2009. Evidence for high levels of vertical transmission in *Toxoplasma gondii*. Parasitology, 136, 1877–1885. 19765335 10.1017/S0031182009990941

[51] Hima K, Houémenou G, Badou S, Garba M, Dossou H-J, Etougbétché J, Gauthier P, Artige E, Fossati-Gaschignard O, Gagaré S. 2019. Native and invasive small mammals in urban habitats along the commercial axis connecting Benin and Niger, West Africa. Diversity, 11, 238.

[52] Houéménou H, Tweed S, Dobigny G, Mama D, Alassane A, Silmer R, Babic M, Ruy S, Chaigneau A, Gauthier P. 2020. Degradation of groundwater quality in expanding cities in West Africa. A case study of the unregulated shallow aquifer in Cotonou. Journal of Hydrology, 582, 124438.

[53] Kulldorff M, Huang L, Pickle L, Duczmal L. 2006. An elliptic spatial scan statistic. Statistics in Medicine, 25, 3929–3943. 16435334 10.1002/sim.2490

[54] Lilue J, Müller UB, Steinfeldt T, Howard JC. 2013. Reciprocal virulence and resistance polymorphism in the relationship between *Toxoplasma gondii* and the house mouse. ELife, 2, e01298. 24175088 10.7554/eLife.01298 PMC3810784

[55] Mariën J, Lo Iacono G, Rieger T, Magassouba N, Günther S, Fichet-Calvet E. 2020. Households as hotspots of Lassa fever? Assessing the spatial distribution of Lassa virus-infected rodents in rural villages of Guinea. Emerging Microbes & Infections, 9, 1055–1064. 32459576 10.1080/22221751.2020.1766381 PMC7336995

[56] Marshall PA, Hughes JM, Williams RH, Smith JE, Murphy RG, Hide G. 2004. Detection of high levels of congenital transmission of *Toxoplasma gondii* in natural urban populations of *Mus domesticus*. Parasitology, 128, 39–42. 15002902 10.1017/s0031182003004189

[57] Mercier A, Devillard S, Ngoubangoye B, Bonnabau H, Bañuls A-L, Durand P, Salle B, Ajzenberg D, Dardé M-L. 2010. Additional haplogroups of *Toxoplasma gondii* out of Africa: Population structure and mouse-virulence of strains from Gabon. PLoS Neglected Tropical Diseases, 4, e876. 21072237 10.1371/journal.pntd.0000876 PMC2970538

[58] Mercier A, Garba M, Bonnabau H, Kane M, Rossi J-P, Dardé M-L, Dobigny G. 2013. Toxoplasmosis seroprevalence in urban rodents: a survey in Niamey, Niger. Memórias do Instituto Oswaldo Cruz, 108, 399–407. 23828008 10.1590/0074-0276108042013002 PMC3970615

[59] Mikhail MW, Hasan AH, Allam KA, Mohammed NM. 2017. Seroprevalence of *Toxoplasma gondii* among commensal rodents from Giza governorate, Egypt. Journal of the Egyptian Society of Parasitology, 47, 145–150. 30157343

[60] Muradian V, Ferreira LR, Lopes EG, Oliveira Esmerini P, Jesus Pena HF de, Soares RM, Gennari SM. 2012. A survey of *Neospora caninum* and *Toxoplasma gondii* infection in urban rodents from Brazil. Journal of Parasitology, 98, 128–134. 21790367 10.1645/GE-2817.1

[61] Murillo-León M, Müller UB, Zimmermann I, Singh S, Widdershooven P, Campos C, Alvarez C, Könen-Waisman S, Lukes N, Ruzsics Z, Howard JC, Schwemmle M, Steinfeldt T. 2019. Molecular mechanism for the control of virulent *Toxoplasma gondii* infections in wild-derived mice. Nature Communications, 10, 1233. 10.1038/s41467-019-09200-2 PMC6420625 30874554

[62] Murphy RG, Williams RH, Hughes JM, Hide G, Ford NJ, Oldbury DJ. 2008. The urban house mouse (Mus domesticus) as a reservoir of infection for the human parasite *Toxoplasma gondii*: an unrecognised public health issue? International Journal of Environmental Health Research, 18, 177–185. 18569146 10.1080/09603120701540856

[63] Owen MR, Trees AJ. 1998. Vertical transmission of *Toxoplasma gondii* from chronically infected house (*Mus musculus*) and field (*Apodemus sylvaticus*) mice determined by polymerase chain reaction. Parasitology, 116, 299–304. 9585931 10.1017/s003118209700231x

[64] Parsons MH, Banks PB, Deutsch MA, Corrigan RF, Munshi-South J. 2017. Trends in urban rat ecology: a framework to define the prevailing knowledge gaps and incentives for academia, pest management professionals (PMPs) and public health agencies to participate. Journal of. Urban Ecology, 3, jux005.

[65] Parsons MH, Banks PB, Deutsch MA, Munshi-South J. 2018. Temporal and space-use changes by rats in response to predation by feral cats in an urban ecosystem. Frontiers in Ecology and Evolution, 146.

[66] Pawełczyk O, Asman M, Solarz K. 2020. The discovery of zoonotic protozoans in fleas parasitizing on pets as a potential infection threat. Acta Parasitologica, 65, 817–822. 32468231 10.2478/s11686-020-00221-2 PMC7679334

[67] Reperant LA, Hegglin D, Tanner I, Fischer C, Deplazes P. 2009. Rodents as shared indicators for zoonotic parasites of carnivores in urban environments. Parasitology, 136, 329–337. 19154652 10.1017/S0031182008005428

[68] Ruiz A, Frenkel JK. 1980. Intermediate and transport hosts of *Toxoplasma gondii* in Costa Rica. American Journal of Tropical Medicine and Hygiene, 29, 1161–1166. 7446807 10.4269/ajtmh.1980.29.1161

[69] Sanchez SG, Besteiro S. 2021. The pathogenicity and virulence of *Toxoplasma gondii*. Virulence, 12, 3095–3114. 34895084 10.1080/21505594.2021.2012346 PMC8667916

[70] Selvin S, Ragland KE, Chien EY-L, Buffler PA. 2004. Spatial analysis of childhood leukemia in a case/control study. International Journal of Hygiene and Environmental Health, 207, 555–562. 15729836 10.1078/1438-4639-00327

[71] Shapiro K, Bahia-Oliveira L, Dixon B, Dumètre A, de Wit LA, VanWormer E, Villena I. 2019. Environmental transmission of *Toxoplasma gondii*: Oocysts in water, soil and food. Food and Waterborne Parasitology, 15, e00049. 32095620 10.1016/j.fawpar.2019.e00049 PMC7033973

[72] Shwab EK, Saraf P, Zhu X-Q, Zhou D-H, McFerrin BM, Ajzenberg D, Schares G, Hammond-Aryee K, van Helden P, Higgins SA, Gerhold RW, Rosenthal BM, Zhao X, Dubey JP, Su C. 2018. Human impact on the diversity and virulence of the ubiquitous zoonotic parasite *Toxoplasma gondii*. Proceedings of the National Academy of Sciences, 115, E6956–E6963. 10.1073/pnas.1722202115 PMC6055184 29967142

[73] Sibley LD, Boothroyd JC. 1992. Virulent strains of *Toxoplasma gondii* comprise a single clonal lineage. Nature, 359, 82–85. 1355855 10.1038/359082a0

[74] Sikes RS, Care A. 2016. Mammalogists UC of the AS of. 2016. Guidelines of the American Society of Mammalogists for the use of wild mammals in research and education. Journal of Mammalogy, 97, 663–688. 29692469 10.1093/jmammal/gyw078 PMC5909806

[75] Simon S, de Thoisy B, Mercier A, Nacher M, Demar M. 2019. Virulence of atypical *Toxoplasma gondii* strains isolated in French Guiana in a murine model. Parasite, 26, 60. 31549631 10.1051/parasite/2019048 PMC6757855

[76] Sirdar MM, Fosgate GT, Blignaut B, Mampane LR, Rikhotso OB, Du Plessis B, Gummow B. 2021. Spatial distribution of foot-and-mouth disease (FMD) outbreaks in South Africa (2005–2016). Tropical Animal Health and Production, 53, 1–12. 10.1007/s11250-021-02807-y 34181093

[77] Skotarczak B. 2016. The role of ticks in transmission cycle of *Toxoplasma gondii*. Annals of. Parasitology, 62, 185–191. 10.17420/ap6203.52 27770758

[78] Stryjek R, Mioduszewska B, Spaltabaka-Gędek E, Juszczak GR. 2018. Wild Norway rats do not avoid predator scents when collecting food in a familiar habitat: a field study. Scientific Reports, 8, 1–11. 29930280 10.1038/s41598-018-27054-4 PMC6013492

[79] Team RC. 2020. A language and environment for statistical computing, 2015. R foundation for statistical computation: Vienna, Austria.

[80] Tenter AM, Heckeroth AR, Weiss LM. 2000. *Toxoplasma gondii*: from animals to humans. International Journal for Parasitology, 30, 1217–1258. 11113252 10.1016/s0020-7519(00)00124-7 PMC3109627

[81] de Thois B, Demar M, Aznar C, Carme B. 2003. Ecologic correlates of *Toxoplasma gondii* exposure in free-ranging neotropical mammals. Journal of Wildlife Diseases, 39, 456–459. 12910778 10.7589/0090-3558-39.2.456

[82] Tonouhewa ABN, Akpo Y, Sessou P, Salanon C, Aplogan GL, Assogba MN, Youssao IAK, Farougou S. 2020. *Toxoplasma gondii* infections in pigs from south Benin and assessment of breeders’ knowledge about toxoplasmosis. Bulgarian Journal of Veterinary Medicine, 23, 248–256.

[83] Tonouhewa ABN, Akpo Y, Sherasiya A, Sessou P, Adinci JM, Aplogan GL, Youssao I, Assogba MN, Farougou S. 2019. A serological survey of *Toxoplasma gondii* infection in sheep and goat from Benin, West-Africa. Journal of Parasitic Diseases, 43, 343–349. 31406398 10.1007/s12639-018-01076-1 PMC6667587

[84] VanWormer E, Fritz H, Shapiro K, Mazet JA, Conrad PA. 2013. Molecules to modeling: *Toxoplasma gondii* oocysts at the human–animal–environment interface. Comparative Immunology, Microbiology and Infectious Diseases, 36, 217–231. 23218130 10.1016/j.cimid.2012.10.006 PMC3779781

[85] Wang X, Dong L, Zhang L, Lv Y, Li Q, Li H. 2019. Genetic characterization of *Toxoplasma gondii* from wild rodents in Sichuan Province, Southwestern China. Iranian Journal of Parasitology, 14, 106–110. 31123474 PMC6511585

[86] Yan C, Liang L-J, Zheng K-Y, Zhu X-Q. 2016. Impact of environmental factors on the emergence, transmission and distribution of *Toxoplasma gondii*. Parasites & Vectors, 9, 137. 26965989 10.1186/s13071-016-1432-6 PMC4785633

[87] Zhou Y, Zhang H, Cao J, Gong H, Zhou J. 2016. Epidemiology of toxoplasmosis: role of the tick *Haemaphysalis longicornis*. Infectious Diseases of Poverty, 5, 1–6. 26897021 10.1186/s40249-016-0106-0 PMC4761159

[88] Zulpo DL, Sammi AS, Dos Santos JR, Sasse JP, Martins TA, Minutti AF, Cardim ST, de Barros LD, Navarro IT, Garcia JL. 2018. *Toxoplasma gondii*: a study of oocyst re-shedding in domestic cats. Veterinary Parasitology, 249, 17–20. 29279081 10.1016/j.vetpar.2017.10.021

