# Supplementary material for: Molecular prevalence, genetic characterization and patterns of Toxoplasma gondii infection in domestic small mammals from Cotonou, Benin
Source: Parasite. 2022 Dec 21;29:58. doi: 10.1051/parasite/2022058 (PMC9879161; doi:10.1051/parasite/2022058)
Supplement: Supplementary file 1 — Supplementary Table 1: Captures and species-specific prevalence by sampled localities and sites [file parasite-29-58-s1.pdf]

Supplementary Table 1 : Captures and species-specific prevalence by sampled localities and sites. “**Rra**”, “**Rno**”, “**Mna**”, “**Mus**”, “**Cro**, “**Cga**” and “**Pde**” stand for *Rattus rattus, Rattus norvegicus, Mastomys natalensis, Mus musculus, Crocidura olivieri*, *Cricetomys gambianus* and *Praomys derooi* respectively; “**Pos**”: qPCR-positive individuals, “**N**”: number of captured animals.

		**All species**		**Rra**		**Rno**		**Mna**		**Mus**		**Cro**		**Cga**		**Pde**	
**Localities**	Sites	N	Pos (%)	N	Pos (%)	N	Pos (%)	N	Pos (%)	N	Pos (%)	N	Pos (%)	N	Pos (%)	N	Pos (%)
**Agla**	a1	12	0 (0)	8	0	-	-	1	0 (0)	-	-	3	0 (0)	-	-	-	-
	a2	8	0 (0)	3	0	-	-	2	0 (0)	-	-	3	0 (0)	-	-	-	-
	a3	5	1 (20)	2	0	1	0 (0)	-	-	-	-	2	1 (50)	-	-	-	-
	a4	6	1 (16.7)	-	-	-	-	-	-	-	-	6	1 (16.7)	-	-	-	-
	a5	28	5 (17.9)	14	2 (14.3)	-	-	8	1 (0.1)	-	-	6	2 (33.3)	-	-	-	-
	a6	11	0 (0)	6	0 (0)	-	-	1	0 (0)	-	-	4	0 (0)	-	-	-	-
	a7	8	1 (12.5)	7	1 (14.3)	-	-	-	-	-	-	1	0 (0)	-	-	-	-
	a8	16	3 (18.8)	4	1 (25)	6	1 (16.7)	-	-	-	-	6	1 (16.7)	-	-	-	-
	a9	3	0 (0)	1	0 (0)	-	-	-	-	-	-	2	0 (0)	-	-	-	-
	a10	10	0 (0)	-	-	4	0 (0)	-	-	-	-	6	0 (0)	-	-	-	-
	**All**	**107**	**11 (10.3)**	**45**	**4 (8.9)**	**11**	**1 (9.1)**	**12**	**1 (8.33)**	**-**	**-**	**39**	**5 (12.8)**	**-**	**-**	**-**	**-**
**Ladji**	l1	9	0 (0)	4	0 (0)	3	0 (0)	-	-	-	-	2	0 (0)	-	-	-	-
	l2	15	3 (20)	3	0 (0)	-	-	-	-	-	-	12	3 (25)	-	-	-	-
	l3	11	2 (18.2)	5	1 (20)	1	0 (0)	-	-	-	-	5	1 (20)	-	-	-	-
	l4	10	0 (0)	7	0 (0)	-	-	1	0 (0)	-	-	2	0 (0)	-	-	-	-
	l5	6	1 (16.7)	2	0 (0)	-	-	-	-	-	-	4	1 (25)	-	-	-	-
	l6	14	3 (21.4)	8	1 (12.5)	-	-	-	-	-	-	6	2 (33.3)	-	-	-	-
	l7	11	1 (9.1)	7	1 (14.3)	-	-	-	-	-	-	4	0 (0)	-	-	-	-
	l8	6	2 (33.3)	-	-	-	-	-	-	-	-	6	2 (33.3)	-	-	-	-
	l9	16	0 (0)	12	0 (0)	-	-	-	-	-	-	4	0 (0)	-	-	-	-
	l10	13	0 (0)	10	0 (0)	-	-	-	-	-	-	3	0 (0)	-	-	-	-
	l11	4	2 (50)	-	-	1	1 (100)	2	1 (50)	-	-	1	0 (0)	-	-	-	-
	**All**	**115**	**14 (12.2)**	**58**	**3 (5.2)**	**5**	**1 (20)**	**3**	**1 (33.3)**	**-**	**-**	**49**	**9 (18.4)**	**-**	**-**	**-**	**-**

Saint-Jean	s1	2	0 (0)	1	0 (0)	-	-	-	-	-	-	-	-	1	0 (0)	-	-
	s2	4	0 (0)	-	-	-	-	-	-	-	-	4	0 (0)	-	-	-	-
	s3	19	0 (0)	19	0 (0)	-	-	-	-	-	-	-	-	-	-	-	-
	s4	13	1 (7.7)	8	1 (12.5)	-	-	-	-	-	-	4	0 (0)	1	0 (0)	-	-
	s5	7	1 (14.3)	2	1 (50)	-	-	-	-	-	-	4	0 (0)	1	0 (0)	-	-
	s6	9	5 (55.6)	1	0 (0)	-	-	-	-	-	-	4	2 (50)	1	1 (100)	3	2 (66.7)
	s7	15	4 (26.7)	3	0 (0)	-	-	4	1 (25)	-	-	5	3 (60)	1	0 (0)	2	0 (0)
	s8	10	3 (30)	-	-	-	-	-	-	-	-	8	3 (37.5)	2	0 (0)	-	-
	s9	5	2 (40)	-	-	-	-	1	0 (0)	-	-	4	2 (50)	-	-	-	-
	s10	3	0 (0)	-	-	-	-	2	0 (0)	-	-	1	0 (0)	-	-	-	-
	**All**	**87**	**16 (18.4)**	**34**	**2 (5.9)**	**-**	**-**	**7**	**1 (14.3)**	**-**	**-**	**34**	**10 (26.5)**	**7**	**1 (14.3)**	**5**	**2 (40)**
APC	p1	73	12 (16.4)	30	3 (10)	-	-	-	-	-	-	43	9 (20.9)	-	-	-	-
	p2	18	2 (11.1)	15	2 (13.3)	-	-	3	0 (0)	-	-	-	-	-	-	-	-
	p3	17	2 (11.8)	11	1 (9.1)	1	0 (0)	-	-	1	0 (0)	4	1 (25)	-	-	-	-
	p4	23	2 (8.7)	-	-	10	0 (0)	-	-	12	1 (8.3)	1	1 (100)	-	-	-	-
	p5	43	13 (30.2)	-	-	12	4 (33.3)	-	-	29	9 (31)	2	0 (0)	-	-	-	-
	p6	46	8 (17.4)	-	-	23	4 (17.4)	-	-	22	4 (18.2)	1	0 (0)	-	-	-	-
	p7	40	5 (12.5)	-	-	13	1 (7.7)	1	0 (0)	24	4 (16.7)	2	0 (0)	-	-	-	-
	p8	36	8 (22.2)	21	5 (23.8)	1	0 (0)	1	0 (0)	12	3 (25)	1	0 (0)	-	-	-	-
	p9	27	3 (11.1)	20	3 (15)	1	0 (0)	1	0 (0)	2	0 (0)	3	0 (0)	-	-	-	-
	**All**	**323**	**55 (17)**	**97**	**14 (14.4)**	**61**	**9 (14.8)**	**6**	**0 (0)**	**102**	**21 (20.6)**	**57**	**11 (19.3)**	**-**	**-**	**-**	**-**
**Total**		**632**	**96 (15.2)**	**234**	**23 (9.8)**	**77**	**11 (14.3)**	**28**	**3 (10.7)**	**102**	**21 (20.6)**	**179**	**35 (19.6)**	**7**	**1 (14.3)**	**5**	**2 (40)**
