# Supplementary material for: Molecular prevalence, genetic characterization and patterns of Toxoplasma gondii infection in domestic small mammals from Cotonou, Benin
Source: Parasite. 2022 Dec 21;29:58. doi: 10.1051/parasite/2022058 (PMC9879161; doi:10.1051/parasite/2022058)
Supplement: Supplementary file 2 — Supplementary Table 2: Prevalence by sex, age, session, and flea carriage. [file parasite-29-58-s2.pdf]

Supplementary Table 2 : Prevalence by sex, age, session and fleas porting. "**Rra**", "**Rno**", "**Mna**", "**Mus**", "**Cro,** "**Cga**" and "**Pde**" stand for *Rattus rattus, Rattus norvegicus, Mastomys natalensis, Mus musculus, Crocidura olivieri*, *Cricetomys gambianus* and *Praomys derooi* respectively; "Pos": qPCR-positive individuals, "**N**": number of captured animals.

			**All species**		**Rra**		**Rno**		**Mna**		**Mus**		**Cro**		**Cga**		**Pde**	
			N	Pos (%)	N	Pos (%)	N	Pos (%)	N	Pos (%)	N	Pos (%)	N	Pos (%)	N	Pos (%)	N	Pos (%)
Agla	Sex	M	49	5 (10.2)	24	2 (8.3)	5	0 (0)	6	0 (0)	-	-	14	3 (21.4)	-	-	-	-
		F	58	6 (10.3)	21	2 (9.5)	6	1 (16.7)	6	1 (16.7)	-	-	25	2 (8)	-	-	-	-
	Age	Ad	82	8 (9.8)	30	2 (6.7)	9	1 (11.1)	10	1 (10)	-	-	33	4 (12.1)	-	-	-	-
		Juv	13	2 (15.4)	10	2 (20)	2	0 (0)	1	0 (0)	-	-	-	-	-	-	-	-
	Fleas	Yes	8	1 (12.5)	3	0 (0)	1	0 (0)	4	1 (25)	-	-	-	-	-	-	-	-
		No	96	9 (9.4)	42	4 (9.5)	10	1 (10)	8	0 (0)	-	-	36	4 (11.1)	-	-	-	-
	Session	S1	50	6 (12)	27	3 (11.1)	5	1 (20)	5	0 (0)	-	-	13	2 (15.4)	-	-	-	-
		S2	57	5 (8.8)	18	1 (5.6)	6	0 (0)	7	1 (14.3)	-	-	26	3 (11.5)	-	-	-	-
Ladji	Sex	M	50	6 (12)	23	1 (4.3)	2	0 (0)	1	0 (0)	-	-	24	5 (20.8)	-	-	-	-
		F	65	8 (12.3)	35	2 (5.7)	3	1 (33.3)	2	1 (50)	-	-	25	4 (16)	-	-	-	-
	Age	Ad	78	10 (12.8)	28	0 (0)	3	1 (33.3)	3	1 (33.3)	-	-	44	8 (18.2)	-	-	-	-
		Juv	27	1 (3.7)	23	1 (4.3)	1	0 (0)	-	-	-	-	3	0 (0)	-	-	-	-
	Fleas	Yes	5	0 (0)	3	0 (0)	1	0 (0)	3	1 (33.3)	-	-	1	0 (0)	-	-	-	-
		No	95	14 (14.7)	53	3 (5.7)	4	1 (25)	-	-	-	-	35	9 (25.7)	-	-	-	-
	Session	S1	52	9 (17.3)	29	3 (10.3)	4	1 (25)	3	1 (33.3)	-	-	16	4 (25)	-	-	-	-
		S2	63	5 (7.9)	29	0 (0)	1	0 (0)	-	-	-	-	33	5 (15.2)	-	-	-	-
Saint-Jean	Sex	M	40	5 (12.5)	15	0 (0)	-	-	5	0 (0)	-	-	15	5 (33.3)	4	0 (0)	1	0 (0)
		F	47	11 (23.4)	19	2 (10.5)	-	-	2	1 (50)	-	-	19	5 (26.3)	3	1 (33.3)	4	2 (50)
	Age	Ad	59	16 (27.1)	12	2 (16.7)	-	-	7	1 (14.3)	-	-	32	10 (31.3)	3	1 (33.3)	5	2 (40)
		Juv	27	0 (0)	22	0 (0)	-	-	-	-	-	-	2	0 (0)	3	0 (0)	-	-
	Fleas	Yes	2	0 (0)	2	0 (0)	-	-	-	-	-	-	-	-	-	-	-	-
		No	79	15 (19)	32	2 (6.3)	-	-	7	1 (14.3)	-	-	28	9 (32.1)	7	1 (14.3)	5	2 (40)
	Session	S1	47	3 (6.4)	26	0 (0)	-	-	5	1 (20)	-	-	10	2 (20)	5	0 (0)	1	0 (0)
		S2	40	13 (32.5)	8	2 (25)	-	-	2	0 (0)	-	-	24	8 (33.3)	2	1 (50)	4	2 (50)

APC	Sex	M	143	22 (15.4)	52	6 (11.5)	23	2 (8.7)	1	0 (0)	49	11 (22.4)	18	3 (16.7)	-	-	-	-
		F	180	33 (18.3)	45	8 (17.8)	38	7 (18.4)	5	0 (0)	53	10 (18.9)	39	8 (20.5)	-	-	-	-
	Age	Ad	262	46 (17.6)	66	11 (16.7)	50	4 (8)	5	0 (0)	92	21 (22.8)	49	10 (20.4)	-	-	-	-
		Juv	39	6 (15.4)	23	1 (4.3)	7	4 (57.1)	1	0 (0)	4	0 (0)	4	1 (25)	-	-	-	-
	Fleas	Yes	66	8 (12.1)	27	3 (11.1)	32	4 (12.5)	-	-	5	0 (0)	2	1 (50)	-	-	-	-
		No	246	46 (18.7)	70	11 (15.7)	24	4 (16.7)	6	0 (0)	95	21 (22.1)	51	10 (19.6)	-	-	-	-
	Session	S1	162	38 (23.5)	35	7 (20)	24	4 (16.7)	3	0 (0)	62	18 (29)	38	9 (23.7)	-	-	-	-
		S2	161	17 (10.6)	62	7 (11.3)	37	5 (13.5)	3	0 (0)	40	3 (7.5)	19	2 (10.5)	-	-	-	-
All localities	Sex	M	282	38 (13.5)	114	9 (7.9)	30	2 (6.7)	13	0 (0)	49	11 (22.4)	71	16 (22.5)	4	0 (0)	1	0 (0)
		F	350	58 (16.6)	120	14 (11.7)	47	9 (19.1)	15	3 (20)	53	10 (18.9)	108	19 (17.6)	3	1 (33.3)	4	2 (50)
	Age	Ad	481	80 (16.6)	136	15 (11)	62	6 (9.7)	25	3 (12)	92	21 (22.8)	158	32 (20.3)	3	1 (33.3)	5	2 (40)
		Juv	106	9 (8.5)	78	4 (5.1)	10	4 (40)	2	0 (0)	4	0 (0)	9	1 (11.1)	3	0 (0)	-	-
	Fleas	Yes	81	9 (11.1)	35	3 (8.6)	34	4 (11.8)	4	1 (25)	5	0 (0)	3	1 (33.3)	-	-	-	-
		No	516	84 (16.3)	197	20 (10.2)	38	6 (15.8)	24	2 (8.3)	95	21 (22.1)	150	32 (21.3)	7	1 (14.3)	5	2 (40)
