# Supplementary material for: Molecular prevalence, genetic characterization and patterns of Toxoplasma gondii infection in domestic small mammals from Cotonou, Benin
Source: Parasite. 2022 Dec 21;29:58. doi: 10.1051/parasite/2022058 (PMC9879161; doi:10.1051/parasite/2022058)
Supplement: Supplementary file 3 — Supplementary Figure: Biplot PCA Cotonou, axis 1 & 2 and 3 & 4. [file parasite-29-58-s3.pdf]

Supplementary figure : PCA Cotonou Biplot, A : axis 1 & 2 ; B : axis 3 & 4.

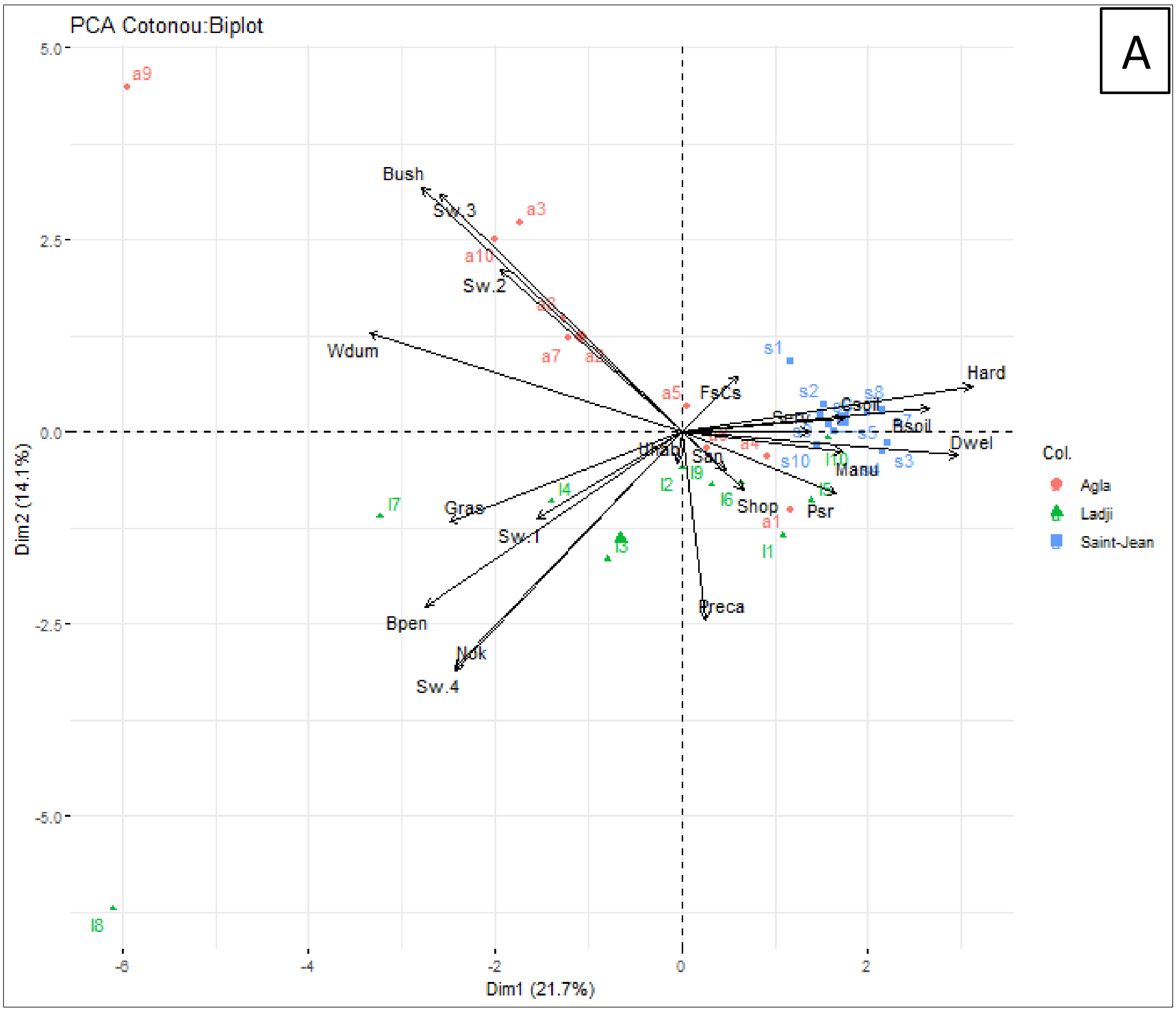

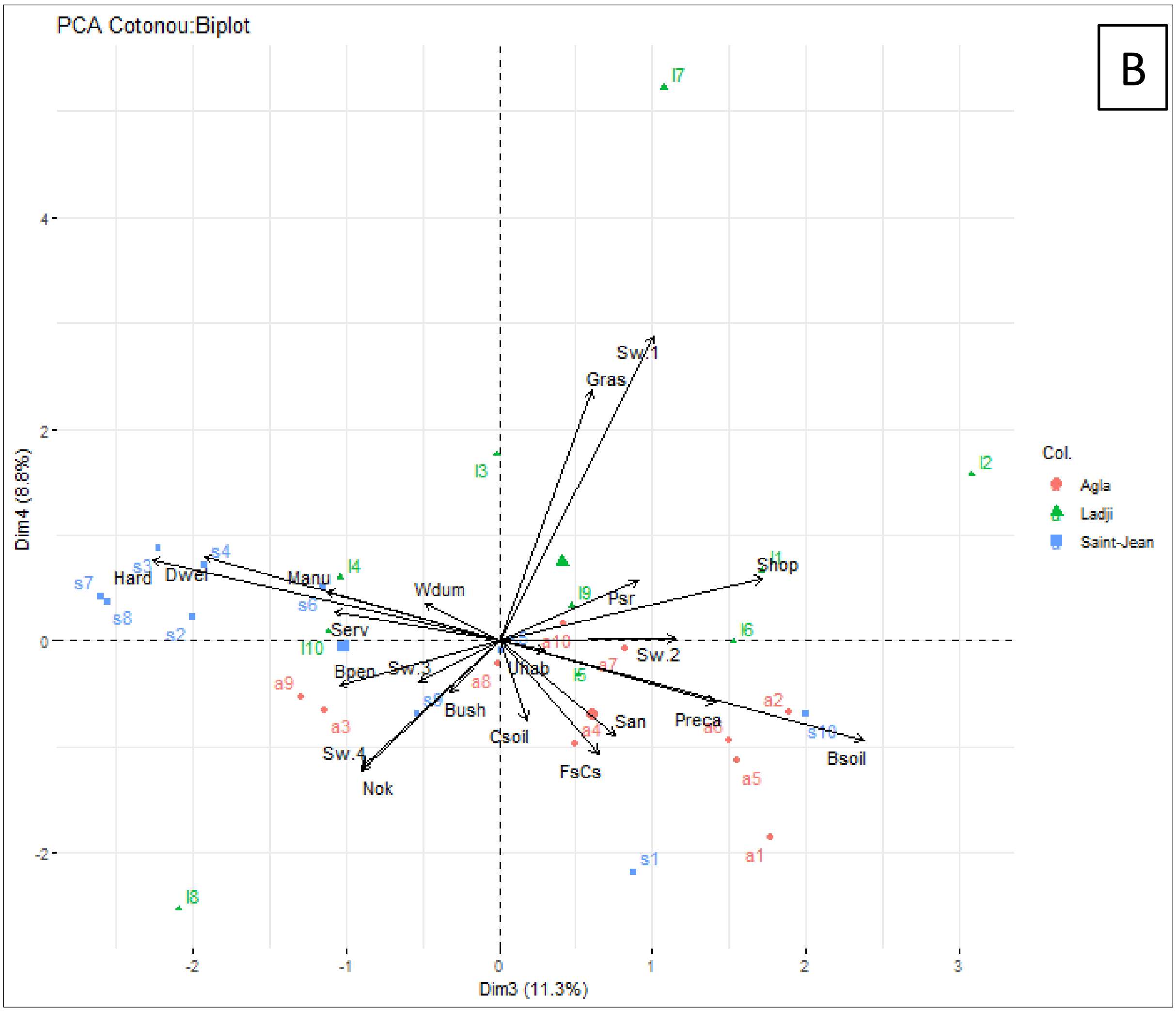

Hard: Hard-built houses; **Preca:** Precarious houses**; Psr:** Precarious spaces and roofed; **Gras**: Grassy-cover; **San**: Sanitation; **Wdum**: Wild dumps; **Nok**: Lake Nokoué; **Csoil**: Cemented soils; **Bsoil**: Bare soil; **Serv**: Services and office; **Dwel**: Dwelling ; **FsCs**: Food storage and cooking space ; **Manu**: Manufacturing space ; **Shop**: Shop with non-food items store ; **Bpen**: Breeding-pens ; **Uhab** : Uninhabited ; **Toil**: Toilet ; **CarS**: Cars station ; **Sw.1 to 4**: Presence of standing water once to four times out of 4 sessions.
